# Unleashing the Impact of Topological Surface States on the Thermoelectric Properties of Granular Sb_2_Te_3_ Thin Films Deposited on Flexible Substrates

**DOI:** 10.1021/acsami.5c03871

**Published:** 2025-06-11

**Authors:** Lorenzo Locatelli, Pietro Rossi, Arun Kumar, Claudia Wiemer, Alessio Lamperti, Roberto Mantovan, Grazia Raciti, Kai Xu, Juan Sebastián Reparaz, Mario Caironi, Giuseppina Pace

**Affiliations:** † Institute for Microelectronics and Microsystems, 9327 National Research Council (IMM-CNR), Via C. Olivetti 2, 20864 Agrate, Italy; ‡ 403543 Center for Nano Science and Technology, Istituto Italiano di Tecnologia, Via Raffaele Rubattino, 81, Milan 20134, Italy; § Department of Physics, Politecnico di Milano, Piazza Leonardo da Vinci 32, Milano 20133, Italy; ∥ Department of Science and Engineering of Matter, Environment and Urban Planning, Università Politecnica delle Marche, Via Brecce Bianche 12, 60131 Ancona, Italy; ⊥ Institut de Ciència de Materials de Barcelona, 16379 ICMAB-CSIC, Campus UAB, 08193 Bellaterra, Spain

**Keywords:** Topological insulators, flexible thermoelectrics, Sb_2_Te_3_, thin film, MOCVD

## Abstract

Between thermoelectric materials, topological insulators (TIs) such as Sb_2_Te_3_ can effectively decouple phonon and electronic transport. Recent works mostly focused on TI composites or superlattices, where the contribution of the topological surface states (TSS) to the thermoelectric properties is overshadowed by other mechanisms such as energy filtering or electronic band reorganization. Here, we investigate efficient thermoelectric Sb_2_Te_3_ polycrystalline thin films deposited on plastic foil. Magneto-transport studies show that the presence of TSS in more granular films is responsible for the 2-orders of magnitude higher electronic conductivity compared to thick films owing to larger crystalline domains (> 100 nm). The prevalence of the bulk states in thick films reduces both their thermal and electronic conductivity; however, they are responsible for an increase in the Seebeck coefficient. Overall, we show that to achieve higher thermoelectric performance of single-component TI films, it is necessary to tune the relative contribution of topological and bulk states. This will potentially allow for the development of cost-effective thermoelectric generators, reducing the complexity of competitive systems based on multicomponent heterostructures.

## Graphical abstract

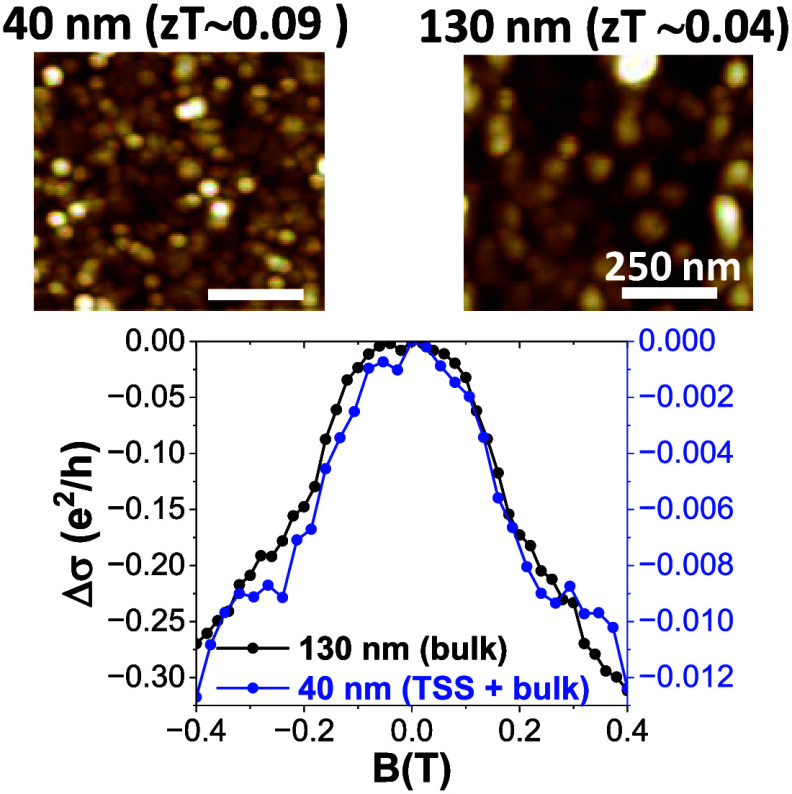

## 1 Introduction

The large density of distributed sensors required for the development of a highly interconnected society has increased the demand for flexible, lightweight, and conformable devices, produced with scalable and low-cost fabrication processes.^1^ However, to enable their sustainable diffusion, they shall be made energy autonomous through their integration with energy harvesters, such as thermoelectric generators. Efficient thermoelectric materials maximize the *zT* factor (eq 1), a dimensionless figure-of-merit which is a function of the thermal conductivity (κ), the electronic conductivity (σ), the Seebeck coefficient (*S*), and the average temperature (*T*) of materials

$$zT=\frac{\sigma S^{2}T}{\kappa }=\frac{PF\times T}{\kappa }$$ 1

where *PF* is the power factor. A major challenge in the optimization of thermoelectric materials is to reduce the mutual dependence of thermal and electric conductivity by decoupling electron and phonon transport.^2^ Topological insulators (TIs), such as Bi_2_Te_3_, Sb_2_Te_3_, and their alloys, are well-known efficient thermoelectric materials.^3–5^ TIs are characterized by bulk states showing a band gap structure and gapless topologically protected surface states (TSS). The electronic transport of the TSS is protected from backscattering caused by non-magnetic disorder and defects; thus, TIs offer a unique opportunity to funnel phonon and electron transport into different channels achieving their effective decoupling,^6^ finally realizing the desired “phonon-glass & electron-crystal” concept.^3,7^ Therefore, with TIs, high heat conversion efficiency can be achieved through the optimization of a single material instead of optimizing complex heterostructures, a unique technological advantage that can simplify fabrication procedures and lower fabrication costs.

Previous works have reported on different approaches to improve the Seebeck coefficient and power factor of Sb_2_Te_3_ films, which mostly rely on materials based on complex heterostructured interfaces as in multilayered^8–10^ or hybrid composites.^11,12^ However, the observed increase in thermoelectric efficiency often depends on the energy filtering effect, where low energy charge carriers are filtered out via backscattering at interfacial barriers, favoring a reduction in thermal conductivity, without sizably compromising the charge carrier mobility. As a result, an increase in *S* is observed, due to its inverse dependence on charge carrier density and to the sharpening of the energy distribution at the Fermi level, along with a lower thermal conductivity leading to an increase in *zT*.^13^

Kim et al. reported on a two-phase system based on the γ-SbTe/Sb_2_Te_3_ composite,^14^ where the carrier energy filtering is considered to lead to an enhanced Seebeck coefficient (*S* ∼ 150 μV/K and *PF* ∼ 10 μW m^–1^ K^–2^, at RT). Similarly, the integration of metallic inclusions, such as the excess Te interphase layer in Sb_2_Te_3_–Te heterojunctions (*PF* ∼ 11.2 μW m^–1^ K^–2^, at RT)^15,16^ or interdomains gold particles,^17^ can promote a lowering of the thermal conductivity while preserving the electronic transport. Hybrid heterostructures, based on Sb_2_Te_3_ nanowires dispersed into an organic^18,19^ or inorganic^20,21^ matrix, allow for achieving efficient phonon scattering but to the expense of electron conductivity. However, the fabrication of such multicompound and multiphase systems is often a more costly strategy, and the influence of nontrivial electronic topology on the thermoelectric properties is often disrupted by chemical and morphological factors, which destroy the spin–orbit coupling and the coherence length of the topological transport vanishing the peculiar properties of TIs.

Recent works have highlighted the role played by the nano/microstructured morphology in bulk TIs in effectively disentangling the phonon and electron transport of thermoelectric films deposited on flexible substrates.^22^ Specifically, while topological surface carriers are more resilient to be transported across different interconnected grains via a common surface transport channel, the phonon transport is limited by the intergrain bottleneck.^23–25^

Here, high thermoelectric performance can be achieved in highly oriented polycrystalline Sb_2_Te_3_ thin films deposited at room temperature on plastic and flexible polyimide (Kapton) substrates via Metalorganic Chemical Vapor Deposition (MOCVD). The low crystallization temperature of Sb_2_Te_3_ (T ≤ 80 °C)^26^ allows the room temperature growth of crystalline films on plastic substrates, a major advantage over other topological insulators, such as Bi_2_Te_3_. MOCVD has already been shown to allow the conformal growth of chalcogenide thin films and nanostructures on large areas, therefore, being compatible with industrial processing and scaling.^27,28^

Despite the simple deposition processing and the low degree of compositional and structural complexity, the resulting films show a PF (188 μW m^–1^ K^–2^ and 378 μW m^–1^ K^–2^ at RT respectively for thin and thick films) competing with the thermoelectric figures of merit of more complex multicomponent and/or multiphase systems.

In thermoelectric materials it is commonly observed that thermal annealing leads to reduced electrical resistivity,^29^ primarily due to the reduction of defect-related scattering as derived from the presence of phonon and/or charge carriers scattering defects.^30^ A similar trend is observed in our study when comparing not-annealed and post-annealed Sb_2_Te_3_ films of the same thickness, confirming the expected decrease in resistivity following annealing. However, when comparing two thermally annealed films of different thicknesses, we find that the thinner but more granular film exhibits significantly higher electrical conductivity than the thicker, more crystalline counterpart. This behavior deviates from the above-mentioned conventional trend, where increased grain size, typically achieved through annealing, correlates with enhanced conductivity due to reduced scattering. The anomalous conductivity enhancement observed in the thinner film is attributed to the key role played by topological surface states (TSS), which can dominate charge transport in these materials. This hypothesis is further supported by magneto-conductance measures, providing evidence of the presence of TI-states in the thin and more granular film.

The presence of the TSS has been investigated by magnetoconductance measurements showing weak antilocalization (WAL) features. We highlight the importance of crystalline grain size and distribution to control the relative contribution of bulk states and TSS to thermoelectric performances. Though showing a reduced *S* compared to thick films, the more granular thin films clearly show a higher contribution of the topologically nontrivial phase to the electronic transport increasing their electronic conductivity (1293 S cm^–1^ in thin films; 622 S cm^–1^ in thick films, at RT). At the same time, the smaller sized crystalline grains found in thin film decrease the thermal conductance, thanks to the presence of a higher density of phonon scattering points at grains’ interconnection. The correlation between morphology, topological transport, and thermoelectric performance in TI thin films was not reported before; nevertheless, this is a fundamental aspect to be exploited before engineering novel complex heterostructured multilayers or composite formulations.

## 2 Results and Discussion

### 2.1 Material Deposition and Characterization

MOCVD was already shown to enable a non-commensurate uniform crystal growth where the stoichiometry of the crystallites is kept as Sb_2.0_Te_3.0_, regardless of the substrate in use or thermal processing.^31^ Here, continuous and uniform films were deposited on a Kapton (polyimide) substrate at room temperature via MOCVD, with a final thickness of 40 and 130 nm (X-ray Reflectivity, XRR data in Figure S1 and Table S1). The structure and crystallinity of the as grown and post-growth annealed (@300 K) Sb_2_Te_3_ films were probed with Grazing Incidence X-ray Diffraction (GIXRD) (Figure 1a and Figure S2). The absence of peaks that are associated with the metallic Te shows that it does not form crystalline inclusions in the films.^32^ Despite the amorphous nature of the polymeric substrates, prevalently crystalline films are obtained already at RT, while the crystalline domains size and their in-plane ordering can be controlled by post-growth annealing (discussion in the SI). Similar conclusions were already reported for Sb_2_Te_3_ films grown on either crystalline or amorphous substrates (such as Si/SiO_2_).^28,31^ X-ray photoelectron spectroscopy (XPS) data are reported in Figures 1c and 1d. The data acquired on the as-grown films (Figure 1c) show an excess of metallic Te (Te–Te, 3d) that is likely to accumulate at the top surface of the films. Upon annealing at 300 °C, both the metallic Te and surface Sb-oxide content is reduced. This is consistent with previous reports showing that excess Te segregates at the top film surface and tends to evaporate during annealing at 300 °C leaving highly stoichiometric crystallites in the films.^33^ Raman spectra (Figure 1b, peaks assignment available in Figure S3) confirm the presence of an excess metallic Te and oxides species in the as grown films, whose presence decreases upon annealing.^34^

**Figure 1:**
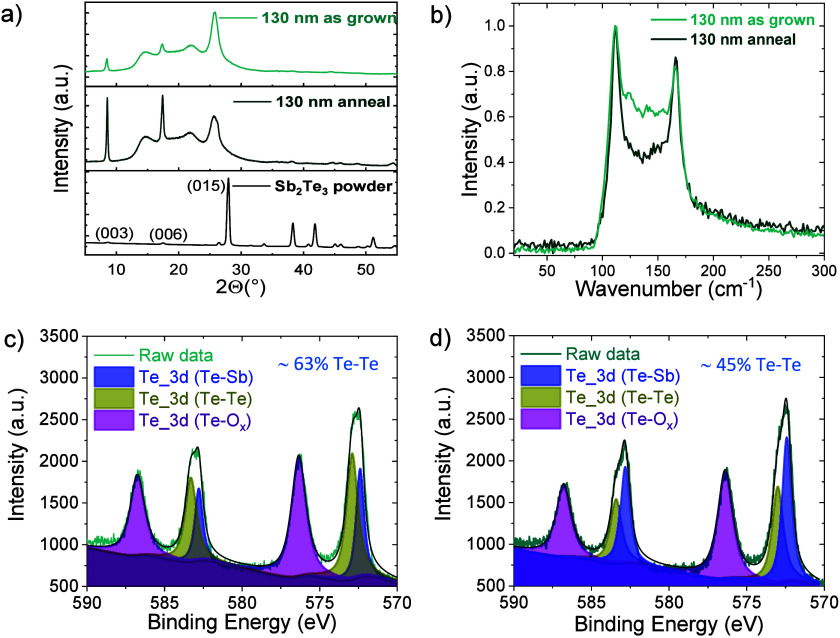
Chemical and morphological analysis of the as grown and annealed Sb_2_Te_3_ film with a thickness of 130 nm. a) XRD pattern. b) Raman spectra. c) and d) XPS spectra of the as grown and annealed film, respectively.

Due to the dependence of the XRD pattern on both grain size and crystallite orientation in our samples, the extrapolation of diffracted domain size is not straightforward from one single diffraction pattern. Therefore, we extracted the grain size distribution from AFM images acquired for each sample before and after annealing (additional SEM images in Figure S4). AFM topography images of the as grown and annealed films are reported in Figure 2a-f. Before thermal treatment (Figure 2a-b), granular domains with tens of nanometers size are already present, which tend to agglomerate to form bigger particles (average diameter ∼ 81 nm). Upon annealing (Figure 2c-f), the highly granular nature of the film is retained. However, analysis of grain size distribution (Figure 2g, raw data counts reported in Figure S5) shows that the thick films are characterized by a strong broadening of the grain’s diameter distribution which is accompanied by a shift in the distribution maximum toward the formation of larger grains (average size 122 nm diameter and large diameter distribution broadening up to 250 nm). Upon annealing of the thin film, a weak broadening of the grains size distribution is observed, that remains centered around ∼ 85 nm, similar to the not-annealed film. The larger grains size of the Sb_2_Te_3_ thick film compared to the thin film is also highlighted in the AFM image profiles reported in Figure 2h.

**Figure 2:**
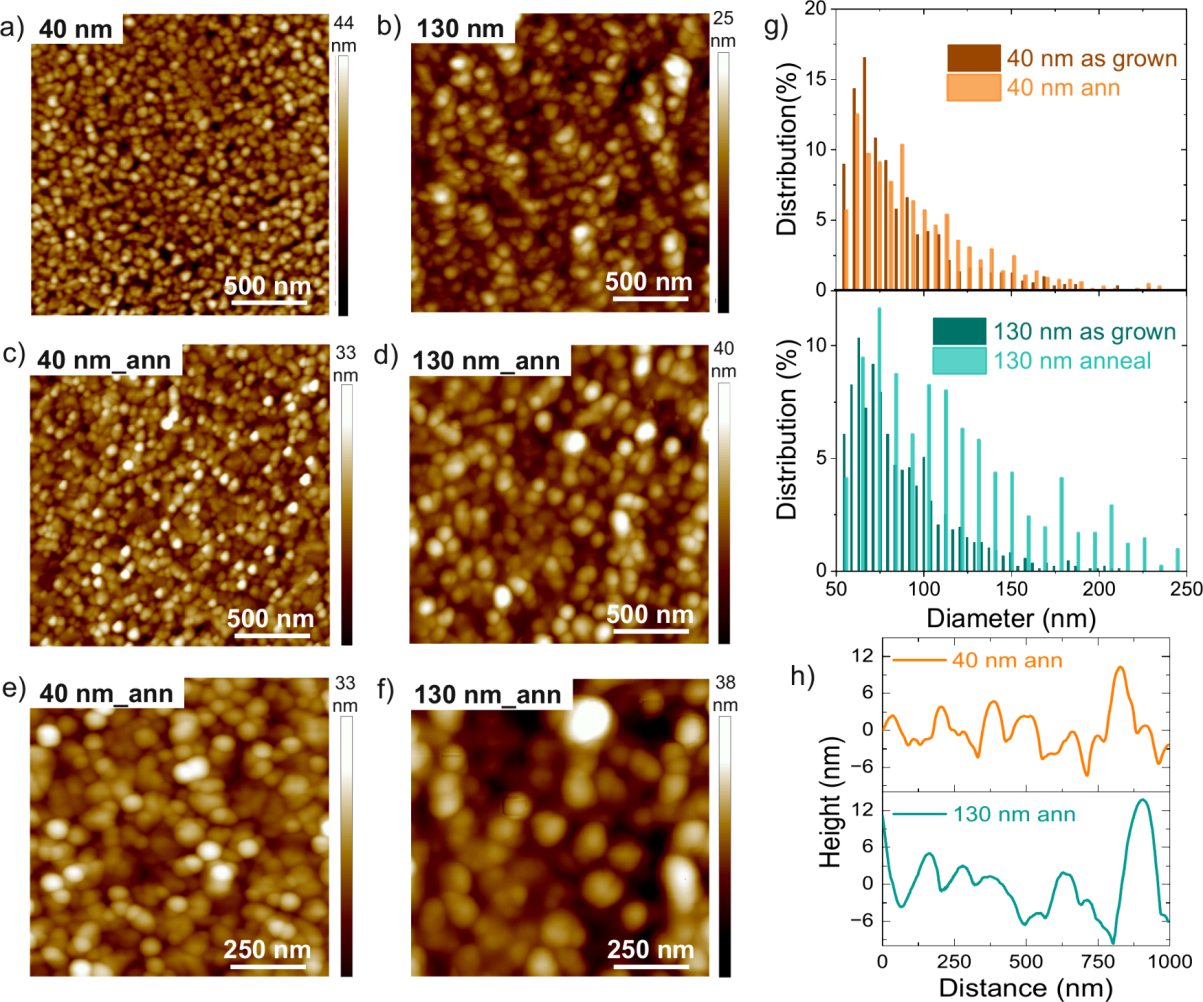
AFM images of the non-annealed films with a thickness of 40 nm (a) and 130 nm (b) and of the annealed films with a thickness of 40 nm (c and e) and 130 nm (d and f). g) Grains diameter distribution measured per each investigated film thickness before and after annealing at 300 °C. h) Line profile showing the higher granularity of the 40 nm thick films compared to the thicker 130 nm film.

Overall, the chemical and structural characterization shows that uniform and polycrystalline Sb_2_Te_3_ films can be obtained also from deposition on an amorphous plastic substrate, with a preferential crystalline orientation of the c-axes perpendicular to the substrate plane. Upon annealing, the film uniformity is preserved, and the crystal size increases with an increase in the density of oriented crystals. A major difference between the 40 and 130 nm annealed films is represented by their different grain size distribution. Specifically, thicker films present larger globular crystals compared to thin films, which instead show a lower surface-to-volume ratio associated with the persistence of smaller grains.

### 2.2 Thermoelectric Characteristics

The improved film crystallinity and higher degree of crystalline orientation obtained after the annealing of both films are responsible for a significant drop in the electrical resistivity (Figure 3a and 3b, Figure S6). An important observation is the clear transition from a thermally activated electronic transport observed in the as-grown films, where the resistivity decreases with temperature, to a semimetallic type of transport found in the annealed films, where the film resistivity increases at higher temperatures.^35,36^ The thin and annealed film shows a room temperature electrical conductivity that is roughly twice that of the thick films (1293 S cm^–1^ and 622 S cm^–1^ respectively, Figure 3c). This behavior is not expected for common polycrystalline material, where the presence of larger grains leads to an improvement of the charge transport performances, and it is a first indication of the concurrent contribution of TSS. The low resistivity achieved after annealing is comparable to that recorded from optimized films grown on Si(111) substrates,^31^ confirming that the commensurate crystal growth is not an essential condition to reach high conductivity in Sb_2_Te_3_.

**Figure 3:**
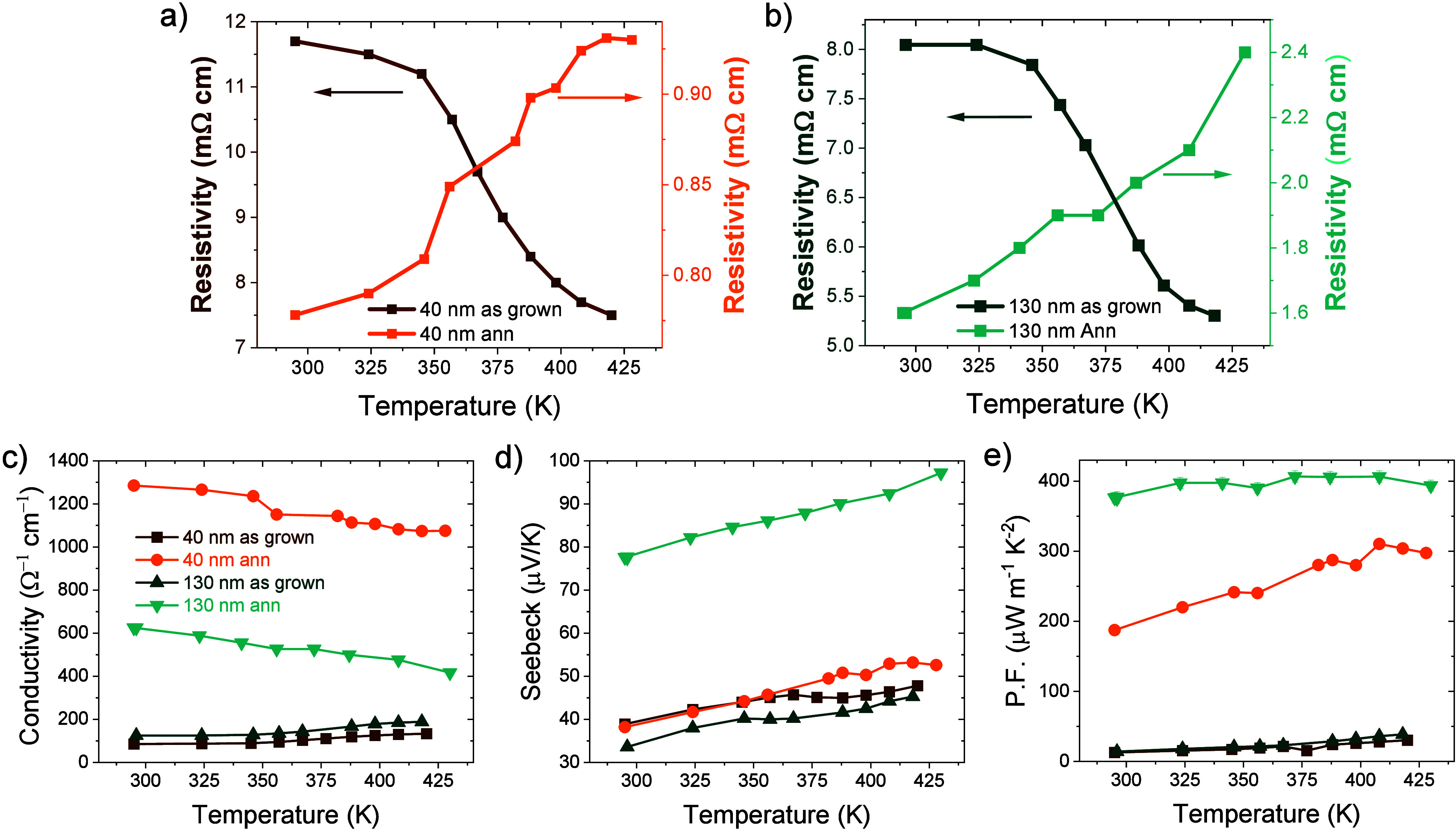
a) and b) Electrical resistivity measured as a function of temperature for the as grown and annealed films with different thicknesses (a) 40 and (b) 130 nm. Temperature dependence of the electrical conductivity (c), Seebeck coefficient (d) and P.F. (e).

The lattice thermal conductivity significantly varies along the crystallographic directions, and for MOCVD grown Sb_2_Te_3_ it was theoretically predicted and experimentally determined to be *k*_*a*‑plane_ = 2.2 W m^–1^ K^–1^ and *k*_*c*‑axis_ = 0.34 W m^–1^ K^–1^ at room temperature.^37^ Since Sb_2_Te_3_ is a highly conductive *p*-type semiconductor, the thermal conductivity can reach up to 7 W m^–1^ K^–1^ in the *a*-plane and from 0.85 to 1.6 W m^–1^ K^–1^ along the *c*-axis, according to the carrier density.^38,39^ Monte Carlo studies showed that to benefit from a reduction in thermal conductivity due to phonon scattering at interfaces, the nanostructure’s size shall be of the order of 100 nm and below, which is the case for the annealed thin film.^11,12^ In the present work, the presence of the low thermal conductivity of the Kapton substrate (κ_kapton_ ∼ 0.1–0.3 W m^–1^ K^–1^) hampers the use of the more common technique, as the 3ω, for the determination of the in-plane (longitudinal) thermal conductivity.^40,41^ However, we measured the cross-plane thermal conductivity (κ_⊥_) measured using frequency-domain thermoreflectance.^42^ The annealed films showed κ_⊥_ values of 4.6 W m^–1^ K^–1^ for the 130 nm and 1.5 W m^–1^ K^–1^ for the 40 nm films. Therefore, due to the larger grain size present in the annealed thick film, the bulk contribution to the thermal properties increases, leading to a rise in the thermal conductivity compared to the thinner film.

The *S* measured at room temperature (295 K) was found to be 38 μV K^–1^ and 78 μV K^–1^ for the annealed thin and thick films, respectively. Considering their different electrical conductivity, the PF (eq 1) resulted in 188 μW m^–1^ K^–2^ and 376 μW m^–1^ K^–2^ (at 295 K) for the annealed thin and thick films (297 μW m^–1^ K^–2^ and 430 μW m^–1^ K^–2^ at 430 K). Such a high PF is competitive or even higher than the one reported for more complex heterostructures.^8,10,15,43,44^ A summary table of the measured thermoelectric data is reported in the Supporting Information (Table S2).

### 2.3 Role of Topological Transport

To assess the role played by the TSS- on the different thermoelectric performances observed in the annealed films, we performed a magneto-transport investigation. In TIs, the presence of a large spin–orbit coupling determines the spin momentum locking, where the spins are transversely locked to the longitudinal crystal momenta. Consequently, a negative magneto-conductance peak is expected at magnetic field (*B*) values close to zero. In Sb_2_Te_3_, the TSS are expected to emerge in crystalline thin films (<50 nm), while in thick films due to the stronger contribution of bulk states, the Fermi level sits within the valence band, and the TI properties are no longer easily manifested in the magneto-transport studies.^26^ The presence of the 2D-type of conduction in nearly epitaxial Sb_2_Te_3_ thin films grown by MOCVD on a crystalline Si-substrate (preannealed at 500 °C) was already attributed to the presence of TSSs following direct visualization of Dirac cones by angle resolved photoemission spectroscopy (ARPES).^45^ Clear weak antilocalization (WAL) effects detected by low-field magneto-transport measurements have also been observed in granular Sb_2_Te_3_ grown on amorphous SiO_2_ by MOCVD.^26^ The presence of TSSwas already reported for polycrystalline and granular films of Bi_2_Se_3_ and Bi_2_Te_3_ and even in amorphous systems.^25,46–49^ However, the correlation between the grain dependent TSS- and the thermoelectric properties of Sb_2_Te_3_ films has not been reported so far. Here, the samples’ sheet resistance (*R_S_*) was measured in the low magnetic field range (*B* = ±0.8 T), and low temperature (∼6–7 K). The sheet magnetoconductance (MC) was calculated as *G_S_* = 1/*R_S_*, and the change in MC (Δσ_s_ = *G_S_* (*B*) – *G_s_* (0)) is reported in Figure 4.

**Figure 4:**
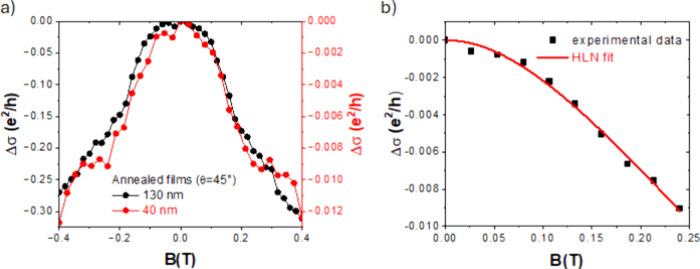
MC measurements. a) Energy normalized MC acquired on the annealed films (*B* oriented at 45° with respect to the sample plane; thickness 40 and 130 nm; 6–7 K) (Lines connecting data points indicate the data trend). b) HLN-fit of the magnetoconductance data acquired from the 40 nm annealed film.

In the as-grown film, a high degree of transport disorder is observed, as anticipated by thermally activated transport (Figure 3a-b). A positive MC is observed, compliant with the presence of localized states and a hopping-like transport (Figure S7).^50,51^ Due to the highly oriented Sb_2_Te_3_ crystallites, the 2D transport prevalently lies parallel to the sample plane, and the contribution of the TSS to the MC is maximized when *B* is oriented perpendicular to the TSS transport channel.^52–55^ However, due to the presence of a polymeric substrate with a thermal conductivity which is lower than the one of the investigated materials (κ_kapton_ ∼ 0.1–0.3 W m^–1^ K^–1^),^56^ the MC measured in a configuration where *B* is perpendicular to the film plane is largely affected by the Ettingshausen effect,^57,58^ which causes distortions and asymmetries of the magnetoconductance curves (Figures S8 and S9). Nevertheless, when measuring in the 45° configuration, we could still detect the TSS influence on the measured MC of thin films while also reducing the spurious thermal effect. The thin annealed film shows a Δσ_s_ that decreases at an increasing magnetic field, and the more cusp-like shape near zero field featuring a WAL shows the presence of strong spin-momentum correlation and high mobility. The MC curves have been analyzed following the Hikami–Larkin–Nagaoka (HLN) theory^59^ (eq 2, eq 3)­

$$\Delta \sigma_{s}=-\alpha \frac{e^{2}}{\pi h}\left[\varphi \left(\frac{1}{2}+\frac{B_{\varphi }}{B}\right)-\ln\left(\frac{B_{\varphi }}{B}\right)\right]+CB^{2}$$ 2

$$B_{\varphi }=\frac{\hbar }{4el_{\varphi }^{2}}$$ 3

where φ­(*x*) is the digamma function, *B*_φ_ is the dephasing field, and *l*_φ_ is the dephasing or coherence length indicating the average distance along which the spin is conserved. The term *CB* ^2^ (*C* is a constant) accounts for the contribution of an electronic transport not driven by quantum confinement phenomena, such as elastic scattering and impurities dependent bulk transport. In the presence of a WAL α is negative and in real samples can cover a continuous range of values reflecting the presence of different contributions to the conduction mechanism. In an ideal 2D-transport regime, α provides information on the number of independent 2D-electronic channels contributing to the conductance. The presence of a topological transport occurring at both top and bottom film surfaces is indicated by a value of α ∼ −1, while a value of α ∼ −0.5 is expected when only one interface is involved (single transport channel). Deviations from such α-values typically reflect the presence of both a 2D and bulk-type electrical transport.

Previous studies have observed a slight kink onset in granular^25^ and porous^60^ samples, which becomes more pronounced in samples with a non-negligible contribution of hopping transport, that we also observe in our thin film after annealing (Figures S8 and S9). This kink marks a transition from topological transport at low magnetic fields to non-topological contributions at higher fields^61^ and is particularly evident in samples with high surface density where hopping transport exceeds bulk diffusive behavior without suppressing topological effects. In the annealed thin films, the best fit is obtained excluding the bulk contribution (*CB* ^2^ term, Figure S10), which provided a value of α ∼ 0.20 and *l*_φ_ ∼ 41 nm, highlighting the relevance of the 2D-transport in the thin film. This is the first experimental evidence of the presence of TSS in granular TI films grown on plastic substrates. The more parabolic shape of the MC measured from the thicker films (SI) is consistent with the presence of a larger contribution of the bulk states compared to the thinner films, and the HLN fitting could not provide straightforward evidence of the presence of 2D-transport. Table S3 summarizes the data extrapolated from the HLN and Hall data analysis.

Therefore, the different MC behavior recorded for the thick and thin films can be explained in terms of the dependence of the relative contribution of TSS and bulk states from their different granular structure and thus their surface-to-volume ratio. The persistence of TSS in amorphous materials was already reported,^62,63^ but the impact of the film granularity on the density of topological states and coherence length was experimentally reported only in highly porous Bi_2_Te_3_.^23,60^ Since the conductivity is almost 2-times higher in thin films (Figure 3), following MC studies, such a large difference is associated with the stronger contribution to the charge transport originated by the TSS compared to thick films. The following discussion elaborates on the impact of such differences in electronic transport over the observed thermoelectric properties.

We reported above that in thick films, the thermal conductivity is higher than in thin films, which according to eq 4 should lead to an increase of *S* as observed

$$S=-\frac{\kappa }{e}\left(\frac{1-b}{1+2b}\right)\left(\frac{E_{0}}{2\mathrm{KT}}\right)+\mathrm{const}$$ 4

where *b* is the hole-to-electron mobility ratio, *K* is Boltzmann’s constant, *e* is the magnitude of the electronic charge, and *E*_0_ is the forbidden band gap at 0 K.^64^ However, at room temperature the ratio of the *S*_thick_/*S*_thin_ (∼2) does not correspond to the ratio of κ_thick_/κ_thin_ (∼3), indicating that an additional energy effect shall be considered. The dependence of *S* on the energy-dependent electrical conductivity σ­(*E*) = *n*(*E*)*qμ*(*E*) at the Fermi energy (*E*_*F*_)^65^ is given by the Mott eq 5 ^66,67^

$$S=\frac{\pi^{2}}{3}\frac{k_{b}}{q}T{\left(\frac{1}{n}\frac{dn\left(E\right)}{dE}+\frac{1}{\mu }\frac{d\mu \left(E\right)}{dE}\right)}_{E=E_{F}}$$ 5

where *q* is the carrier charge, *μ*(*E*) is the mobility, *n*(*E*) = *g*(*E*)*f*(*E*) is the carrier density at the energy level *E*, with *f*(*E*) being the Fermi function. According to eq 5, an increase in *S* can be due to (i) an increase in the dependence of the mobility on carrier energy or to (ii) an increase in the energy dependence of *n*(*E)*, as given by an increase in the density of states (*g*(*E*)) over a narrow energy range around *E*_*F*_ (increased chemical potential at *E*_*F*_).^67^ A strong variation of *μ*(*E*) with *E* can occur, for instance, by a scattering mechanism that strongly depends on the energy of the charge carriers. However, both annealed films show a semimetallic transport, and for degenerate semiconductors, *S* mostly depends on the density of states as reported in eq 6 ^67^

$$S=\frac{8\pi^{2}{k_{b}}^{2}}{3eh^{2}}m^{*}T{\left(\frac{\pi }{n}\right)}^{2/3}$$ 6

where m* is the effective mass. While *n* and *μ* in the annealed thick films remain almost invariant with temperature variation, the density of states in the thin films increases by a factor of 1.6 at room temperature (*n*_p,thin_ = 7.6 × 10^19^ cm^–3^; *n*_p,thin_ = 12 × 10^19^ cm^–3^ at 6 K, summary in Table S3). This suggests that the large crystalline grains in thick films contribute to a sharpening of the energy density at *E_F_*, contributing to the observed higher *S*_thick_ compared to thin films. We also observe that *S*_thick_ is almost twice *S*_thin_ (*S*_thick_ ∼ 2*S*_thin_), which reflects their charge density difference (*n*_Hal,thin_ ∼ 2×*n*_Hal,thick_; *n*_p,thick_ = 3.7 × 10^19^ cm^–3^ at RT, SI) in line with the expected results of eq 6. Since the difference in Hall mobility would provide an opposite dependence (at 6 K, μ_Hal,thick_ ∼ 3 μ_hal,thin_; μ_Hal,thick_ = 164 cm^2^ V^–1^ s^–1^; μ_Hal,thin_ = 54.5 cm^2^ V^–1^ s^–1^), the energetic contribution causing the higher value of *S*_thick_ as compared to *S*_thin_ mostly relies on a narrowing of the *g­(E)* at the *E_F_* in thick films. This is in line with the presence of larger crystalline domains in thick films providing longer range order, and with previous theoretical prediction of Hinsche *et* al.,^68^ showing an inverse dependence of *S* with the carrier density in the presence of a charge transport controlled by TSS. When considering the measured thermal conductivity, the *zT* is found to be ∼0.04 for the thin film and ∼0.02 for the thick film (at 430 K ∼ 0.09 and ∼ 0.04 respectively). Therefore, while the *S* and PF are found to be higher in the thick film, the thin film can lead to a higher *zT* factor, demonstrating to be a more ideal configuration for thermoelectric applications due to its lower thermal conductivity and higher electronic conductivity.

## 3 Experimental Section

### Materials Growth

Polycrystalline Sb_2_Te_3_ thin films were grown at room temperature (RT) on flexible substrates (polyimide, Kapton Dupont, 50 μm thick) with an AIXTRON 200/4 MOCVD reactor operating with ultrapure nitrogen carrier gas. Electronic grade antimony trichloride (SbCl_3_) and bis­(trimethylsilyl)­telluride (Te­(SiMe_3_)_2_) were used as metal–organic precursors. The optimized growth conditions and partial pressures of the two precursors were 2.23 × 10^–4^ mbar and 3.25 × 10^–4^ mbar for SbCl_3_ and Te­(SiMe_3_)_2_ with a total flow of 5.575 L min^–1^, deposition pressure 15 mbar (90 min). The reactor pressure and temperature were 50 mbar and 27 °C respectively. Postgrowth annealing of the Sb_2_Te_3_ films was performed at 300 °C in situ in the MOCVD chamber or in a furnace, under N_2_ atmosphere for 15 min.

### Chemical Characterization

Confocal micro-Raman spectroscopy was performed using a Renishaw In-Via spectrometer (New Mills, Kingswood, Wotton-under-Edge, UK) equipped with a solid-state laser source and excitation wavelength of 514 nm (2.41 eV). The laser power was set below 1% of its nominal power (*<*=1 mW) to avoid sample damage. X-ray Photoelectron Spectroscopy (XPS) data were collected on a PHI 5600 instrument (monochromatic Al Ka X-ray source, 1486.6 eV) equipped with a concentric hemispherical analyzer. The spectra were acquired at a 45° takeoff angle. The spectra were referenced to the C 1s signal set at 284.8 eV. The Te and Sb 3d and 4d spectra were recorded.

### Structural Characterization

The structural-chemical X-ray analysis was performed by means of Grazing Incidence X-ray Diffraction (GIXRD), a Cu–K_α_ line (λ = 1.54 Å), and a position-sensitive gas detector (Inel CPS-120). The rocking angle between the sample and the X-ray beam was fixed at ω = 2°. The thickness of the thin film samples was estimated by X-ray reflectivity (XRR) acquired on glass substrates. The thickness for the thick film was extracted from SEM cross section images.

### Surface Morphology Characterization

Atomic Force Microscopy (AFM) images were acquired in tapping mode with a Dimension Icon, Bruker, USA and using sharp silicon AFM probes (TESPA, Bruker). Scanning Electron Microscopy (SEM) images were acquired on a ZEISS Supra 40 with an acceleration voltage of 15 kV.

### Thermal Characterization

The cross-plane thermal conductivity (along the direction perpendicular to the film plane, κ_⊥_) was measured by using a frequency-domain thermoreflectance. This is a non-destructive contactless optical method where an amplitude-modulated pump laser beam is used to heat the sample (pump, 405 nm) and a probe beam (probe, 532 nm) senses the local temperature at the surface of a sample.^69^ The phase lag between the pump and probe lasers was measured as a function of the modulation frequency of the pump, which was subsequently modeled using the diffusive heat equation. As a result, the thermal conductivity of the sample can be obtained.

### Electrical Characterization and Magnetoresistance

Resistivity (ρ) and magneto-conductance (MC) measurements were carried out on the Sb_2_Te_3_ thin films by using homemade setups. The resistivity (ρ) was acquired in the four points probe (van der Pauw) configuration on ∼1 × 1 cm^2^ samples and in vacuum. The sheet magnetoresistance (*R_S_*, where *R_S_* = ρ/*t* and *t* is the film thickness) was measured as a function of the magnetic field (*B*) and temperature (*T*) in the four points probe van der Pauw configuration and under high vacuum. A constant current of 100 mA was applied to the samples with a Keithley 2610, and the voltage changes were measured during the magnetic field scan with a nanovoltmeter (Keithley 2182A). The magneto-conductance (*G_S_*) was calculated from the inverse of the *R_S_* (*G_S_* = 1/R_S_) and was measured over magnetic fields up to ±0.8 T and a variable orientation of the *B* vector with respect to the surface plane (3°, 45°, and 90°). The HLN fitting is performed after averaging positive and negative fields in the reduced ±0.25 T range. For data comparison, the baseline corrected and energy normalized *G_S_* was reported (Δσ, eq 7)­

$$\Delta \sigma =\frac{G_{S}\left(B\right)-G_{S}\left(0\right)}{e^{2}/h}$$ 7

where *G_S_* (0) is the value of *G_S_* at zero field, *e* is the electron charge (*e* = 1.6 × 10^–19^ C), and *h* is the Planck constant.

The Hall mobility (μ_*Hal*_) and carrier density (*n*) were calculated from the measured resistivity (ρ) and Hall effect signal according to eqs 8 and 9

$$n=\frac{IB}{teV_{H}}$$ 8

$$\mu_{Hal}=\frac{1}{enR_{S}}$$ 9

where *V_H_* is the Hall voltage, *t* is the thickness of the film, *R_S_* is the sheet resistance, and *B* is the magnitude of the applied magnetic field. The nature of the carriers (holes or electrons) was deduced by evaluating the slope of the recorded Hall voltage in a *V*_*H*_(*B*) graph.

We also highlight the influence of the Ettingshausen effect on the WAL experiments that is caused by the poor thermal conductivity of plastic substrates, adding spurious contribution to the magneto-conductance. Due to the low thermal conductivity of our Kapton substrates, heat dissipation in the sample is poor, and thermal effects, and specifically the Ettingshausen effect, more strongly interfere in the magneto-conductance measurements. The contribution of such an effect (*V_E_*) to the measured voltage is linearly proportional to the Ettingshausen coefficient (*P*, *V_E_* ∝ *P*). The Ettingshausen coefficient for metals is of the order of 10^–10^ K A^–1^ m, while for semimetals such as WTe_2_ and Bi, due to their poor thermal conductivity, *P* is of the order of 10^–4^.

### Thermoelectric Characterization

The in-plane Seebeck coefficient was measured using a custom-built setup optimized for thin film characterization, using a quasi-static approach.^70^ The measurements were conducted under vacuum conditions (10^–4^ mbar), in the temperature range from RT to 150 °C compatible with the inner limitation of the setup. According to the setup requirements, the samples were deposited following the described MOCVD protocol on Kapton substrates (1.5 × 1.5 cm) with two parallel gold contacts deposited with an electron-beam evaporator and patterned using a shadow mask (thickness = 30 nm, interelectrode distance = 5 mm). Measurements were performed over 2–3 samples per each type of thermal treatment and thickness. Thermoelectric data as reported in Figure 3 of the main text showing additional experimental error bars are reported in the SI.

## 4 Conclusion

We show that the highly ordered structure and the TSS typically observed in Sb_2_Te_3_ thin films are retained on an amorphous plastic substrate. We demonstrated that by varying the thin film grain size it is possible to control the prevalent contribution of either the bulk states or TSS on the thermoelectric properties. Due to their topological nature, TSS contribution to the electronic transport prevails in highly granular films (average grain size <85 nm), where a high electron conductivity and low thermal conductance are achieved. When the average grain size >100 nm, the charge carrier transport is mostly ruled by the bulk states, providing a higher Seebeck coefficient but at the expense of a lower charge conductivity and higher thermal conductivity compared to more granular films. The difference in the Seebeck coefficient is explained in terms of a higher degeneracy of the semimetallic bulk states associated with larger crystalline grains which reduce the charge carrier density at the Fermi level. This work shows that high thermoelectric performance, that competes with the one reported for more complex heterostructured thermoelectric materials, can be achieved in single component TI thin film, largely simplifying materials and fabrication processing.

## Supplementary Material



## Notes

Additional experimental data on chemical films, structural and morphological characterization; summary tables of thermoelectric and magnetotransport parameters; additional magnetotransport data and analysis (PDF)
