# Supplementary material for: Unleashing the Impact of Topological Surface States on the Thermoelectric Properties of Granular Sb2Te3 Thin Films Deposited on Flexible Substrates
Source: ACS Appl Mater Interfaces. 2025 Jun 11;17(25):37206–15. doi: 10.1021/acsami.5c03871 (PMC12203476; doi:10.1021/acsami.5c03871)
Supplement: Supplementary file 1 [file am5c03871_si_001.pdf]

Supporting Information

Unleashing the Impact of Topological Surface States on the Thermoelectric Properties of Granular Sb_2Te_3 Thin Films Deposited on Flexible Substrate

Lorenzo Locatelli,[a,†] *Pietro Rossi,*[b,c,†] *Arun Kumar,*[d] *Claudia Wiemer,*[a] *Alessio Lamperti,*[a] *Roberto Mantovan,*[a,*] *Grazia Raciti,*[e] *Kai Xu,*[e] *Juan Sebastián Reparaz,*[e] *Mario Caironi,*[b] *Giuseppina Pace* [a,b,*]

L. Locatelli, A. Kumar, C. Wiemer, A. Lamperti, R.Mantovan, G. Pace
[a] Institute for Microelectronics and Microsystems, National Research Council (IMM-CNR), Via C. Olivetti 2, 20864 Agrate, Italy

L. Locatelli, C. Wiemer, A. Lamperti, R.Mantovan, G. Pace
[a] Institute for Microelectronics and Microsystems, National Research Council (IMM-CNR), Via C. Olivetti 2, 20864 Agrate, Italy

P. Rossi, M. Caironi, G. Pace
[b] Center for Nano Science and Technology, Istituto Italiano di Tecnologia, Via Raffaele Rubattino, 81, Milan 20134, Italy
P. Rossi
[c] Department of Physics, Politecnico di Milano, Piazza Leonardo da Vinci 32, Milano, 20133, Italy

A.Kumar,
[d] Faculty of Engineering Polytechnic University of Marche, Via Brecce Bianche 12, 60131, Ancona, Italy

[e] Kai Xu, G. Raciti, M. Campoy-Quiles, J. S. Reparaz
Institut de Ciència de Materials de Barcelona, ICMAB-CSIC, Campus UAB, 08193, Bellaterra, Spain

[†] These authors contributed equally.

***** E-mail: giuseppina.pace@cnr.it

***** E-mail: roberto.mantovan@cnr.it

1. Structural and morphological characterization

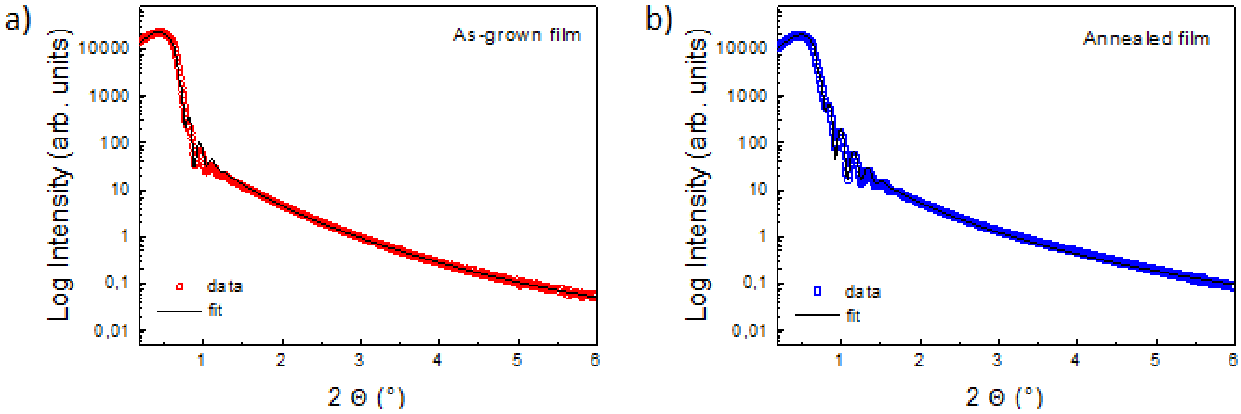

Figure S1: X-ray Reflectivity (XRR) acquired on thin samples grown on glass substrates before (a) and after annealing (b).

Table S1: XRR parameters.

Sb2Te3	Thickness (nm)	Electron density (e/Å^3)	Roughness (nm)
**As-grown**	43.0	1.62	3.5
**Annealed (@300 K)**	40.9	1.69	2.6

Compared to the powder XRD pattern, the as grown and annealed samples show more intense peaks at 2θ = 8.46° and 17.3° corresponding to the 003, 006 reflections which are attributed to the out-of-plane 00*l* oriented rhombohedral crystalline structure in the R*3m* space group. The relatively small linewidths of the (00*l*) peaks indicate larger crystalline size found in the post-growth annealed films accompanied by a higher degree of orientation of the crystallites versus the sample plane. The broader peak at ~ 28.26° is assigned to the 015 reflection, while the broad background between 2θ = 15-30° originates from the scattering of the Kapton substrate. The (015) peak typically shows high intensity in powder diffractogram, and highlights the polycrystalline nature of the film, while its broadening indicates the presence of structural amorphous components.[1] The decrease of the relative intensity of the 015 reflection, along with the higher intensity of the 003 and 006 reflections occurring upon annealing, confirm the increase in crystalline domain size and the preferential orientation of their c-axis perpendicular to the substrate. The persistence of the (015) peaks also after the annealing highlights the presence of residual disorder due to randomly oriented crystallites

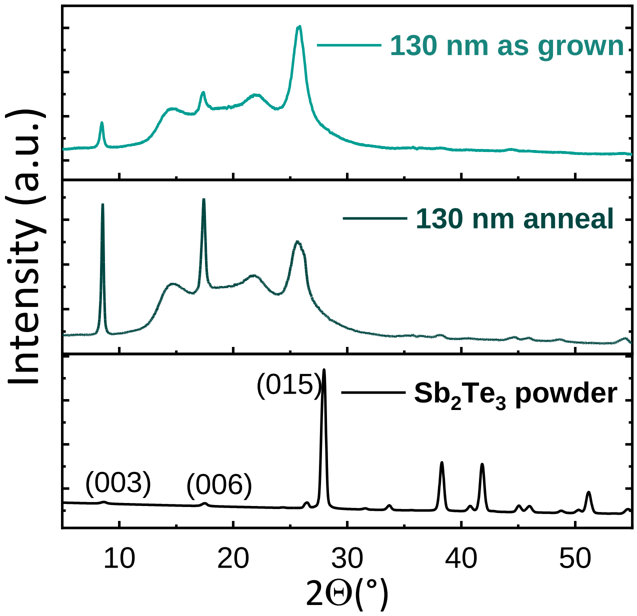

Figure S2: XRD spectra of the as grown and annealed Sb_2Te_3 films with a thickness of 130 nm.

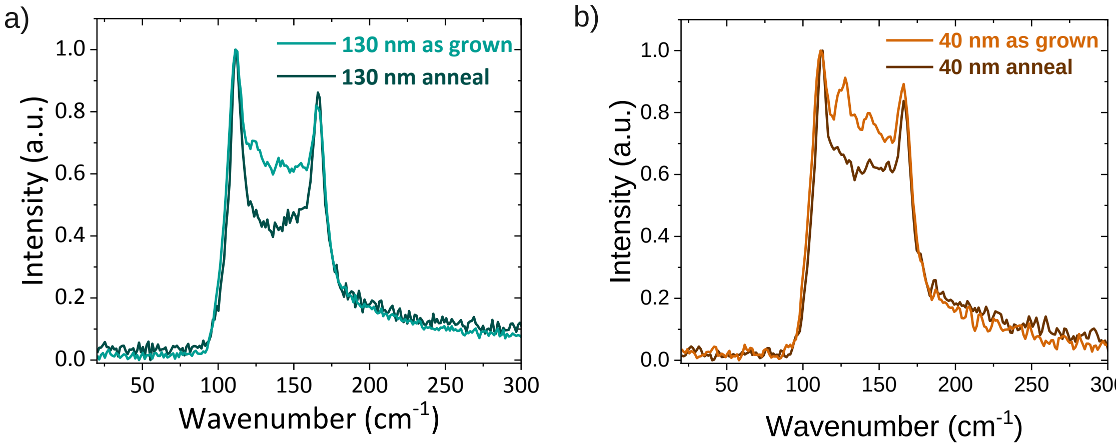

Figure S3: Normalized Raman spectra acquired on the as grown and annealed Sb_2Te_3 films with a thickness of a) 130 nm and b) 40 nm.

In Figure S3, the peak centered at 167-168 cm^{-1} is assigned to A_{1g}^2 phonon modes of the Sb-Te in-plane vibrations, while the peak at 111 cm^{-1} is associated to the Sb-Te in plane E_{1g}^2 mode.[2] Upon annealing at 300 °C, the small shift to higher energy (lower cm^{-1}) of the A_{1g}^2 phonon mode, along with the E_{1g}^2 and A_{1g}^2 peaks sharpening, highlight the occurrence of bond hardening following the removal of disordered regions and the reduced contribution of the excess Te-Te (A^1 (Te-Te) 124 cm^{-1}, E^2 (Te-Te) 140 cm^{-1}) and Sb-O (Sb-O-Sb between 120 cm^{-1} and 150 cm^{-1}).[3]

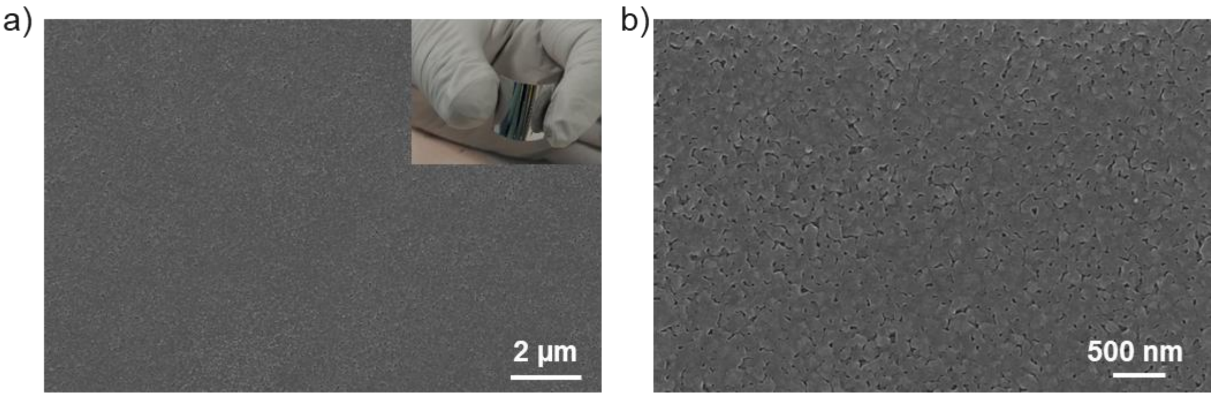

Figure S4: a) SEM images of post-annealed Sb2Te3 (40 nm thick) deposited by MOCVD on plastic substrates. b) Magnified SEM image.

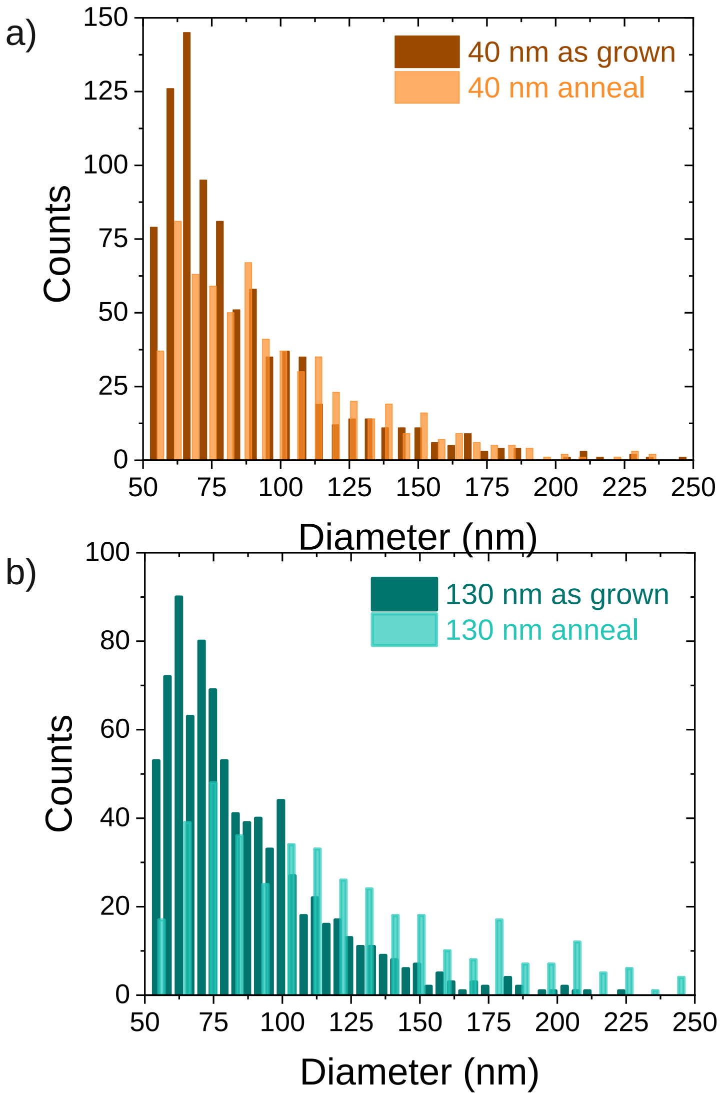

Figure S5: Distribution of grain diameters before and after the annealing treatment, derived from AFM images analysis for a) the 40 nm thick film and b) the 130 nm thick film.

2. Thermoelectric Characterization

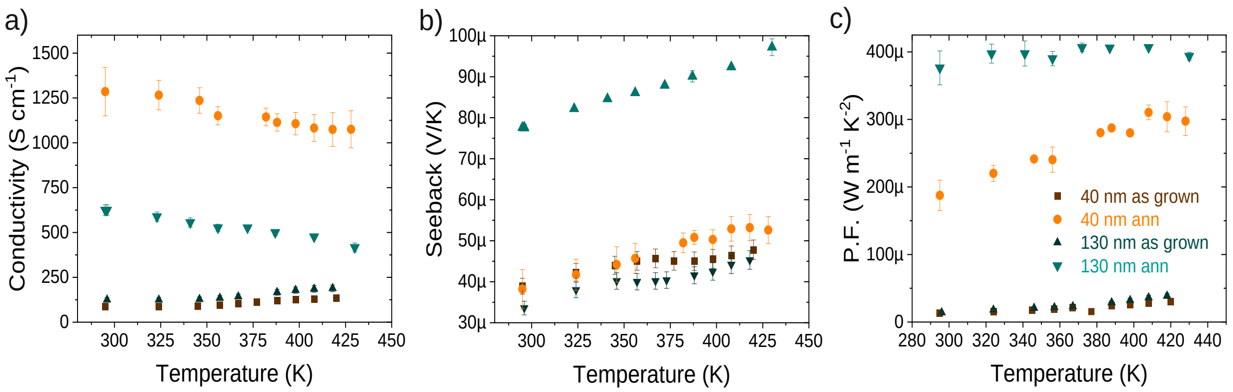

Figure S6: Thermoelectric data as reported in Figure 3 of the main text showing additional experimental error bars. a) Conductivity; b) Seebeck Coefficient; d) power factor (P.F.).

Table S2: Summary of thermoelectric data.

	**40 nm Ann**	**130 nm Ann**
**Thermal conductivity (W m^{-1} K^{-1})**	1,5	4,6
**Electrical conductivity (S/cm)**	1293 (@ 295 K)<br>1074 (@ 430 K)	622 (@ 295 K)<br>410 (@ 430 K)
**Seebeck Coefficient (µV/K)**	38 (@ 295 K)<br>53 (@ 430 K)	78 (@ 295 K)<br>97 (@ 430 K)
**Power Factor (µW m^{-1} K^{-2}) @ 295 K**	188	376
**Power Factor (µW m^{-1} K^{-2}) @ 430 K**	298	394
**ZT @295 K**	0,037	0,024
**ZT* @430 K**	0,085	0,037

*Estimate from thermal conductivity measured at room temperature

3. Magnetoelectric characterization

The spurious thermal artifact, originated from the Ettingshausen effect, was also present in thick films as expected. However, due to their larger heat dissipation volume compared to thin films, its contribution to the MC was less detrimental than for thin films. Therefore, we could also investigate the MC of the as grown (Figure S7) and annealed (Figure S8) thick film. Figure S7, shows the presence of a strong localization compatible with the thermally activated transport already identified in the temperature dependence of the resistance reported in the main text. The strong overlap detected around zero field, between the MC acquired at 3° and 90° (Figure S8), shows how the contribution of the bulk states is prevalent in the thick annealed films.

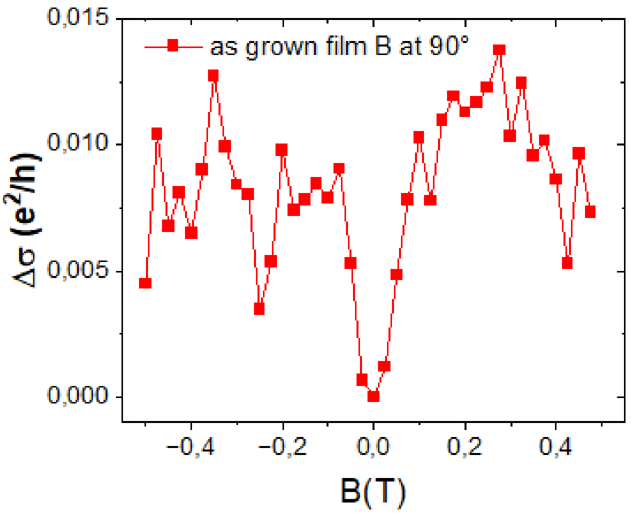

Figure S7: Localized states typically originating thermally induced electron transport (hopping like transport) (130 nm as grown film).

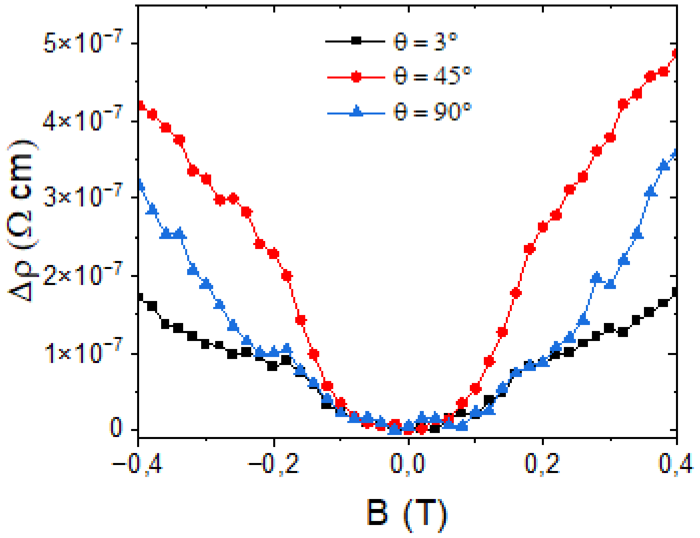

Figure S8: Angle dependent magnetoresistance acquired on thick annealed samples at various orientation of B with respect to the sample plane.

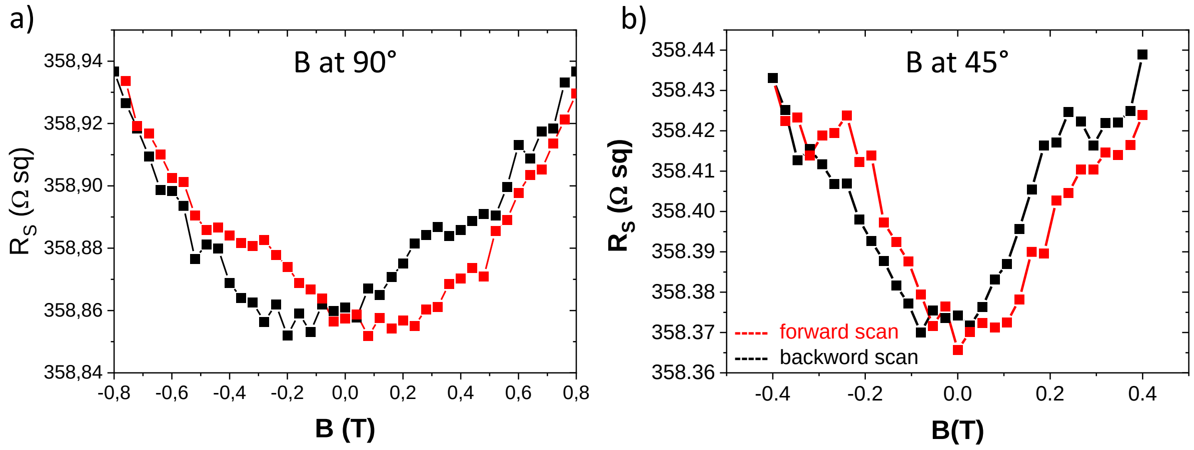

Figure S9: Magnetotransport measurements acquired on annealed thin films (40 nm). a) Backward and forward spectra acquired with magnetic field oriented at 90° with respect to sample plane showing spurious thermal effects. b) Backward and forward spectra acquired with magnetic field oriented at 45°.

Figure S9-a shows the impact of the thermal spurious Ettingshausen effect, that is affecting the magnetotransport measurements acquired on thin films when the magnetic field is oriented perpendicularly to the sample plane (θ=90°). When B is oriented at 45° the influence of the thermal artifact is reduced, and a cusp-like shape emerges (Figures S9-b). The average of the forward and backward spectra shown in Figure 4 has been used to fit the experimental data with the HLN model (data reported in Figure 4 main text).

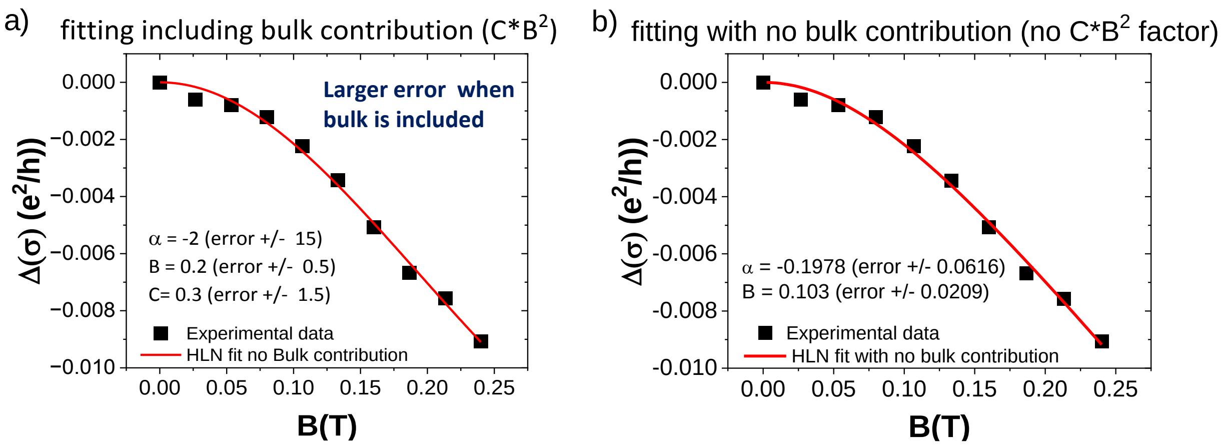

Figure S10: a) HLN fitting including bulk contribution (additional parabolic term $C \cdot B^2$). b) HLN fit where the bulk term is excluded (C = 0), significantly reducing the error bars on the extracted parameters.

Due to the residual influence of the Ettingshausen effect (still present, though reduced, in the 45° configuration) and the presence of the kink feature, we considered the fitting within the ±0.25 T magnetic field range to be more reliable. Importantly, a possible bulk contribution was also accounted for during the fitting process, modeled as an additional parabolic term ($C \cdot B^2$) in the HLN equation (Figure SI-9(a)). Figure SI-9(b) shows a comparison fit where the bulk term is excluded (C = 0), which significantly reduces the error bars on the extracted parameters (α and B → L_ϕ), supporting our interpretation of the MR data as arising from 2D-type conduction.

Table S3. Summary of relevant data extracted from magnetoconductance measurements.

	**α**	**lφ [nm]**	**n_p** [a] **[cm^{-3}] @ 295.3 K**	**μ_{hall}** [b] **[$cm^2\ V^{-1}\ s^{-1}$] @ 295.3 K**	**n_p [cm^{-3}] @ 6 K**	**μ_{hall} [$cm^2\ V^{-1}\ s^{-1}$] @ 6 K**
**130 nm as grown**	----	----	2.35E+19 (θ=90°, 275.72K)	40.5 (θ=90°, 275.72K	2.5E+19 (θ=90°) 3.3E+19 (θ=45°)	45.5 (θ=90°) 35.6 (θ=45°)
**130 nm anneal**	----	----	3.7E+19 (θ=90°, 271 K)	224.5 (θ=90°, 271 K)	θ=45°, 5.25e+19 θ=90°, 3.75E+19	θ=45°, 164 θ=90°, 229
**40 nm anneal (θ=45°)**	α= - 0.20 ± 0.06;	l_Φ = 40.6	7.6E+19	54.51	1.2E+20	50

[a](n_p, carrier density); [b] (μ_{hall}, Hall Mobility)

Variations in charge carrier density and Hall mobility measured at room temperature and at 6-7 K for the as-grown films, fall within the error bar, therefore the ruling charge transport mechanism over the different temperatures must remain the same, and features a hopping like transport.

References

(1) Rimoldi, M.; Cecchini, R.; Wiemer, C.; Lamperti, A.; Longo, E.; Nasi, L.; Lazzarini, L.; Mantovan, R.; Longo, M. Epitaxial and Large Area Sb2Te3thin Films on Silicon by MOCVD. *RSC Adv* **2020**, *10* (34), 19936–19942. https://doi.org/10.1039/d0ra02567d.

(2) Cecchi, S.; Dragoni, D.; Kriegner, D.; Tisbi, E.; Zallo, E.; Arciprete, F.; Holý, V.; Bernasconi, M.; Calarco, R. Interplay between Structural and Thermoelectric Properties in Epitaxial Sb2+xTe3 Alloys. *Adv Funct Mater* **2019**, *29* (2). https://doi.org/10.1002/adfm.201805184.

(3) Mertens, J.; Kerres, P.; Xu, Y.; Raghuwanshi, M.; Kim, D.; Schön, C. F.; Frank, J.; Hoff, F.; Zhou, Y.; Mazzarello, R.; Jalil, A. R.; Wuttig, M. Confinement-Induced Phonon Softening and Hardening in Sb2Te3 Thin Films. *Adv Funct Mater* **2024**, *34* (1), 2307681. https://doi.org/10.1002/adfm.202307681.
